# Expert consensus on the burden of respiratory syncytial virus disease and the utility of nirsevimab for disease prevention and protection of infants

**DOI:** 10.1007/s12519-025-00926-2

**Published:** 2025-06-28

**Authors:** Daniel Y. T. Goh, Anne Goh, Ching Kit Chen, Si Min Chan, Poh Choo Khoo, Koh Cheng Thoon, Jiahui Li, Bee Wah Lee, Chee Fu Yung

**Affiliations:** 1 Division of Paediatric Pulmonary Medicine and Sleep, Khoo Teck Puat National University Children’s Medical Institute, National University Hospital, Singapore, Singapore; 2 Department of Pediatrics, Yong Loo Lin School of Medicine, National University of Singapore, Singapore, Singapore; 3 Allergy and Respiratory Medicine Service, Department of Paediatrics, KK Women’s and Children’s Hospital, Singapore, Singapore; 4 Paediatrics Academic Clinical Programme, SingHealth Duke-NUS Academic Medical Centre, Singapore, Singapore; 5 Division of Cardiology, Department of Paediatrics, Khoo Teck Puat-National University Children’s Medical Institute, National University Health System, Singapore, Singapore; 6 Division of Paediatric Infectious Diseases, Khoo Teck Puat-National University Children’s Medical Institute, National University Hospital, Singapore, Singapore; 7 Department of Neonatology, KK Women’s and Children’s Hospital, Singapore, Singapore; 8 Duke National University of Singapore Medical School, Singapore, Singapore; 9 Lee Kong Chian School of Medicine, Nanyang Technology University, Singapore, Singapore; 10 Infectious Disease Service, KK Women’s and Children’s Hospital, Singapore, Singapore

**Keywords:** Consensus, Infants, Monoclonal antibody, Nirsevimab, Respiratory syncytial virus

## Abstract

**Background:**

Respiratory syncytial virus (RSV) is the most common cause of pediatric acute lower respiratory tract infection worldwide. In Singapore, RSV substantially contributes to the morbidity and mortality of children aged < 5 years, particularly during their first year of life, with hospitalization rates peaking during epidemic outbreaks. This expert consensus paper aims to provide an overview of the RSV disease burden, unmet needs, and the urgency for RSV prevention in all infants.

**Data sources:**

A comprehensive literature review was conducted using Medline via PubMed, to identify relevant studies, including randomized controlled trials, observational studies, systematic reviews, and meta-analyses, related to RSV burden and prevention and nirsevimab. A multidisciplinary group of physicians with RSV expertise from leading institutions in Singapore reviewed the literature on RSV-related topics, convened to deliberate and formulate evidence-based recommendations, summarizing the overall disease burden, unmet needs, and the optimal implementation of an immunization strategy for all infant protection against RSV infections in Singapore. Premeeting and in-meeting surveys were conducted to guide the development of final consensus recommendations.

**Results:**

Epidemiology and burden of RSV in Singapore, current protection against RSV infections in infants, implementations of nirsevimab, and optimization of nirsevimab implementation in Singapore were discussed in this study. Although RSV brings substantial burden with underestimated costs, palivizumab is the only approved product for RSV prevention in Singapore. Existing evidences reveal that nirsevimab has a good safety profile and is effective in preventing RSV lower respiratory tract infections. Seven statements were formulated on the epidemiology and burden of RSV in Singapore, with emphasis on its high incidence and associated healthcare cost, and the clinical efficacy and safety of nirsevimab as a basis of its implementation for all infant protection in Singapore.

**Conclusions:**

The burden of RSV disease in young children is substantial, especially in those < 2 years old, accounting for up to 47% of bronchiolitis and pneumonia admissions in children < 6 months. The administration of a single dose of nirsevimab may be offered to infants at birth for the prevention of RSV in Singapore.

**Graphical Abstract:**

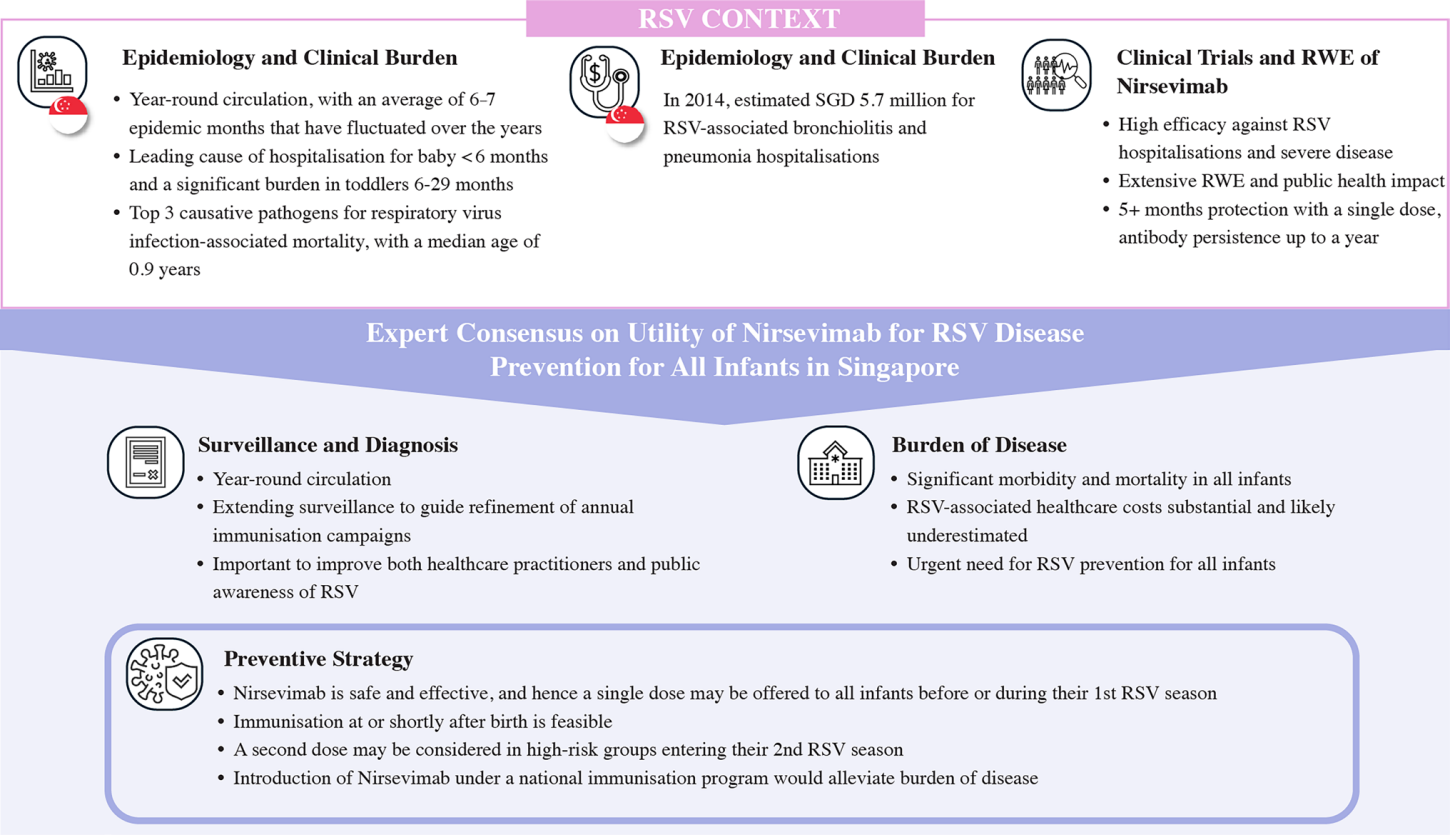

**Supplementary Information:**

The online version contains supplementary material available at 10.1007/s12519-025-00926-2.

## Introduction

Respiratory syncytial virus (RSV), identified in 1956, is the leading cause of respiratory illness in infants and children [1, 2]. According to the World Health Organization, RSV accounts for over 60% of acute respiratory tract infections globally [3]. RSV infections range from mild upper respiratory tract issues to severe bronchiolitis and pneumonia that pose life-threatening risks [1, 4]. The estimates indicate that RSV is responsible for 60%–80% of infant bronchiolitis and up to 40% of pediatric pneumonia cases [1]. In 2019, RSV caused 33 million lower respiratory tract infection (LRTI) episodes, 3.6 million hospital admissions, and 101,400 deaths in children aged 0–60 months globally [5]. The infants aged 28 days to 6 months are at the highest risk, with RSV contributing to one in every 28 deaths in this age group [5]. Other vulnerable groups include children with risk factors like preterm birth, congenital heart disease, and chronic lung disease [6].

RSV from the *Pneumoviridae* family, is an enveloped, single-stranded, negative-strand RNA virus with two subtypes: A and B [2, 7–9]. The virus has 10 genes encoding 11 proteins, including three transmembrane glycoproteins: attachment protein (G), fusion protein (F), and small hydrophobic protein (SH) [7, 9]. The F and G glycoproteins are crucial for viral entry and membrane fusion, forming primary targets for neutralizing antibodies [8]. The G glycoprotein has the highest genetic diversity, enabling the segregation of viral sequences into subtypes A and B, each of which contain multiple genotypes [2]. F is a type I viral fusion protein, with majority of the protective antibodies targeting this protein [2, 9].

RSV is primarily transmitted through respiratory droplets and contact with contaminated surfaces [8, 10]. Natural immunity to RSV is insufficient, leading to frequent reinfections, with the primary episode being the most severe [6, 11]. The health impact of RSV extends beyond respiratory issues, as hospitalized children with RSV bronchiolitis often exhibit low lung function and high rates of allergic sensitization [12]. Additionally, current data show early-life RSV LRTI to be associated with recurrent wheezing and a high incidence of asthma [13–16]. Globally, RSV contributes significantly to morbidity and mortality in children under five and imposes a significant economic burden [5]. A systematic review and meta-analysis of 41 studies from 1987 to 2017 estimated the annual global cost for managing RSV-LRTIs in children under five at about EUR 5 billion [17].

RSV is a seasonal virus, with outbreaks typically occurring from November to April/May in the Northern Hemisphere and from May to September in the Southern Hemisphere, often coinciding with the rainy season in tropical regions [18, 19]. A modeling study by Staadegaard et al. defined RSV seasonal epidemics as the minimum number of epidemic months accounting for at least 75% of annual RSV cases reported [20]. The study reported a median epidemic duration of 10–17 weeks (from December/January to February/March) and 11–15 weeks (from May/June to August/September) in the Northern and Southern Hemispheres, respectively. However, less well-defined epidemics were noted in the (sub)tropics and tropical regions near the equator [20].

The INFORM-RSV study, a multicenter epidemiological study, revealed that RSV subtypes co-circulate with alternating dominance annually [21]. Another study conducted during 2007–2015 in about 4000 hospitalized children with pneumonia in China showed a shift in dominance from RSV-A to RSV-B every two years [22]. The COVID-19 pandemic temporarily reduced RSV circulation due to public health measures, followed by a resurgence as restrictions were eased [10]. This resurgence was accompanied by longer than usual RSV seasonal peaks as reported by several countries, but a recent study showed that RSV activity is returning to pre-pandemic patterns [23–27]. Importantly, children younger than two years old who were not exposed to RSV during the COVID-19 pandemic due to reduced circulation of the virus, formed a RSV-naïve infant cohort with potentially greater susceptibility to RSV [23].

Until 2022, palivizumab, a humanized monoclonal antibody, was the only available immunoprophylaxis for severe RSV disease [28]. Nirsevimab is the first long-acting monoclonal antibody that was recently approved by the US Food and Drug Administration (FDA), the European Union (EU), and Australia for the prevention of RSV in infants < 2 years old [28]. This expert consensus paper aims to discuss the overall RSV disease burden, the unmet needs, and the urgency for RSV prevention in all infants in Singapore. In addition, this paper also provides expert consensus recommendations and position on the utility of nirsevimab for RSV protection in all infants in Singapore.

## Methods

### Expert panel

A multidisciplinary group of nine physicians composed of specialists in pediatrics, respiratory medicine, allergy, cardiology, neonatology, infectious disease and public health from leading academic hospitals and private medical centers across Singapore were identified in view of their relevant clinical expertise in RSV infections.

### Consensus building

The consensus-building process involved a modified Delphi approach [29]. Three core themes were identified: (1) the epidemiology and burden of RSV in Singapore; (2) the usefulness of nirsevimab against RSV infections based on its pivotal trials and real-world studies; and (3) the optimal implementation of nirsevimab in an immunization program.

A comprehensive literature search was performed in Medline via PubMed using search strings developed from a combination of relevant medical subject headings and free-text terms. Studies [randomized controlled trials (RCTs), observational studies, systematic reviews, and meta-analyses] related to nirsevimab, conducted in humans, with abstracts in English, were considered for inclusion, with no year filter (Supplementary Table 1). The shortlisted studies were reviewed and analyzed to develop draft statements. The quality of evidence supporting each recommendation was assessed based on the Oxford Levels of Evidence 2011 (Oxford Centre for Evidence-Based Medicine), summarized in Supplementary Table 2.

The draft statements underwent an iterative editing process and were refined based on real-word clinical experience from the expert panel. In the first round of Delphi voting (Supplementary Table 3), the experts independently rated the statements on a Likert scale (1: completely agree to 5: completely disagree). Consensus (1: completely agree plus 2: agree with minor changes) was set a priori at ≥ 70% agreement [30]. The draft statements along with the summarized literature supporting these statements and the Delphi round 1 results were presented in an advisory board meeting on January 13, 2024. During this meeting, the expert panel discussed and further refined the statements that did not achieve consensus during the first voting. A second survey was conducted during the meeting on the revised draft statements, to inform the development of a total of seven final statements.

The final consensus rating of the statements among the expert panel is summarized in Table 1. The strength of the consensus was defined as “strong” (> 90% agreement), “moderate” (70%–90% agreement), and “weak or no consensus” (< 70% agreement). All statements except one were rated as “strong” recommendations with 100% consensus (Table 1).

**Table 1:** Expert recommendations and consensus rating

Numbers	Statements	Agreement rating	Strength of recommendation	Evidence rating
1	RSV infections follow a peak seasonal pattern from May to September with constant circulation throughout the year in Singapore, reflecting year-round exposure of children to the virus	100%	Strong	Level 2
2	RSV significantly contributes to morbidity and mortality among all infants in Singapore and RSV burden to healthcare systems and child health is underestimated	100%	Strong	Level 2
3	RSV diagnosis has gaps in terms of surveillance, testing, and awareness that need to be addressed	100%	Strong	Level 2
4	There is an urgent need for improved epidemiological surveillance and prevention of RSV infections in all infants in Singapore	88.9%	Moderate	Level 2
5	Nirsevimab has a good safety profile and is effective in preventing RSV LRTIs and hence may be administered to all infants in Singapore before or during their first RSV season	100%	Strong	Level 1
6	Based on the burden of disease, nirsevimab clinical profile, and experience from other countries, RSV immunization should be considered for all infants under the national immunization program in Singapore	100%	Strong	Level 5, expert opinion
7	While the necessity of a second dose of nirsevimab for all infants beyond the first year remains uncertain, administration may be considered in high-risk groups like premature infants	100%	Strong	Level 5, expert opinion

*LRTI* lower respiratory tract infection, *RSV* respiratory syncytial virus

## Recommendations and discussion

### Epidemiology and burden of RSV in Singapore

Four statements were developed on RSV seasonality, burden of disease, and unmet needs related to RSV diagnosis and prevention in Singapore (Table 1, Statements 1–4).

RSV circulates year-round in Singapore, with a seasonal peak occurring during the rainy season between May and September (Table 1, Statement 1; Level of evidence 2) [31–34]. Previously, a global RSV surveillance study which included 601,425 RSV cases from 12 countries defined the RSV season as the minimum number of epidemic months that accounted for at least 75% of annual RSV cases [20]. By applying this definition in previously reported local epidemiological studies, Singapore has an average of 6–7 epidemic months, with a peak typically between May and July, except in 2013. It is, however, notable that the duration of epidemic months and their periodicity have fluctuated over the years [35]. Similar seasonal peak patterns have been noted in other regions. In Thailand and Malaysia, RSV peaks typically occur during the rainy season from July to September [36, 37]. In Hong Kong, China, which experiences tropical to temperate conditions, RSV peaks coincide with higher relative humidity from July to September [38]. Taiwan Province of China also experiences year-round RSV circulation with peaks in August/September [39]. In Queensland, Australia, RSV seasonality has historically been associated with rainfall patterns in tropical regions; however, the recent surveillance data indicate a shift toward year-round circulation, with seasonal peaks from May to September. This shift highlights the importance of ongoing monitoring to capture evolving RSV trends [40, 41]. While the data on RSV seasonality in Singapore is supported by Level 2 evidence, variability in epidemic months over the years suggests the need for continuous surveillance. The studies are largely retrospective, and future prospective studies could help confirm seasonal trends more accurately.

**Fig. 1:**
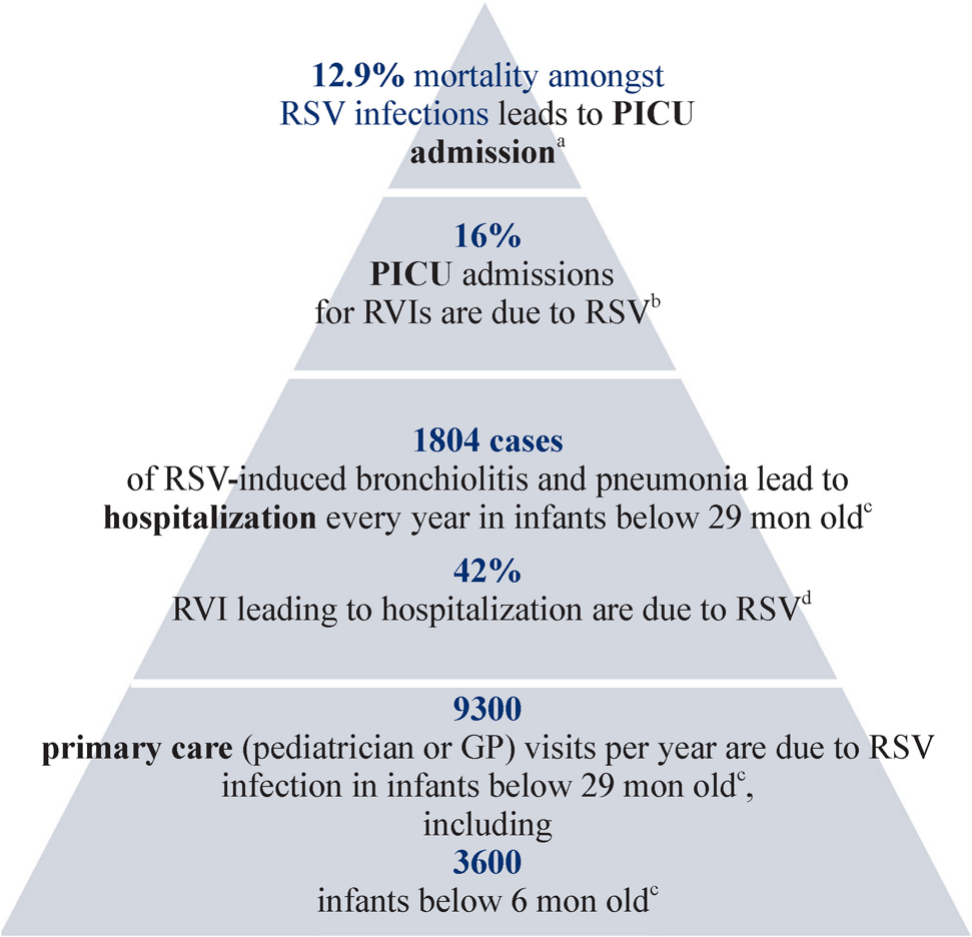
Evidence of RSV significantly contributing to morbidity and mortality among all infants in Singapore. **a** The original study is in Reference [33]. **b** The original study is in Reference [42]. **c** The original study is in Reference [31]. **d** The original study is in Reference [32]. *RSV* respiratory syncytial virus, *PICU* pediatric intensive care unit, *RVI* respiratory viral infection, *GP* general practitioner

RSV significantly contributes to morbidity and mortality among all infants in Singapore (Table 1, Statement 2; Level of evidence 2) (Fig. 1). A community-based study conducted in Singapore showed that among 8436 specimens with flu-like symptoms between 2014 and 2018, there was a 5.8% positivity rate for RSV, with the highest RSV positivity (10.9%) reported in infants ≤ 2 years old [34]. The predominant RSV antigenic subtype was A, except in the year 2016 when RSV type B was predominantly circulating [34]. A statistical and economic model revealed that RSV accounted for 33.5 hospitalizations/1000 child-years among children aged < 6 months and 13.2 hospitalizations/1000 child-years in children aged 6–29 months in Singapore [31]. This represented 47.0% of bronchiolitis and pneumonia admissions in children < 6 months and 34.3% in children 6–29 months, with annual admissions due to RSV-associated bronchiolitis ranging from 135 to 340 for children aged < 6 months and from 271 to 680 for children aged 6–29 months [31]. A separate retrospective analysis of pediatric hospitalizations for respiratory viral infections in a large tertiary women’s and children’s hospital in Singapore demonstrated a positive detection rate of 23.8% among 97,840 specimens, with RSV accounting for 42% of positive results leading to pediatric emergency department visits [32]. Similarly reflecting the high burden of disease, a separate study in the same setting from 2010 to 2019 in a 16-bed pediatric intensive care unit (ICU) reported that respiratory virus infection-associated mortality accounted for 19.8% of all-cause mortality. RSV (16.0%) was among top three causative pathogens identified in these ICU patients after influenza (22.7%) and adenovirus (17.3%) in the study. The median age of respiratory viral infection–associated mortality was 0.9 years [42]. Another study on RSV mortality found that prematurity, hemodynamically significant congenital heart disease [odds ratio (OR) 12.2], immunodeficiency (OR 71.4), and metabolic disease (OR 71.4) were independent risk factors for mortality in hospitalized RSV infections [33]. While the high burden of RSV among all infants in Singapore is supported by Level 2 evidence, it is important to consider the nature of the studies. Much of the RSV hospitalization and mortality data come from KK Women’s and Children’s Hospital, Singapore’s largest pediatric referral center managing a substantial proportion of the country’s pediatric hospitalizations, hence the reported RSV burden is likely representative of the broader population. Future population-based surveillance studies or multi-center cohort studies are needed to confirm RSV’s actual burden in Singapore more accurately.

Gaps in RSV surveillance, testing, and education need to be addressed (Table 1, Statement 3; Level of evidence 2)***.*** The panel discussed that systematic testing and surveillance of RSV is needed to detect the true burden of disease on the healthcare system in Singapore. Currently, data have largely been reported only from a single institution, representing about 57% of RSV-related hospitalizations in infants [32]. There is also a lack of information and surveillance in the primary care setting. Data obtained from local surveillance will allow for comparisons with other countries, especially with a tropical climate, for the implementation of an RSV immunization program and aid adjustments to the healthcare policy in Singapore. Additionally, the panel agreed that awareness on RSV prevention and burden of disease is generally low and often less highlighted compared to other pediatric viruses such as influenza, both among primary healthcare practitioners and members of the public.

To date, only one study has investigated the economic burden of RSV in young children in Singapore. The study showed that the costs of RSV-associated bronchiolitis and pneumonia hospitalizations among children < 30 months totaled Singapore dollar 5.7 million (US dollar 4.3 million) in 2014, with patients covering 60% of expenses. The annual cost of primary care consultations was Singapore dollar 0.46 million (US dollar 0.34 million), with 38% incurred for children aged < 6 months [31]. However, the study highlighted that the economic cost of RSV may have likely been underestimated in view of the high clinical burden. The limitations of the analysis included exclusion of indirect costs (e.g., caregiver productivity loss), lack of data on RSV cases in older children and private pediatric consultations, conservative estimates for private hospital bills. Additionally, the absence of hospital-specific cost data may underestimate the financial burden. In agreement, the expert panel discussed that estimating current RSV-associated healthcare costs may be challenging because the cost of supportive treatment, hospital stay, type of ward, and subsidies provided may be difficult to estimate, and over the years these costs have increased. Moreover, they agreed that rising healthcare costs, work absenteeism, and opportunity costs further contribute to an underestimated RSV burden in Singapore. This scenario emphasizes the importance of optimizing surveillance and preventing RSV infections to reduce overall complications and healthcare costs (Table 1, Statement 4; Level of evidence 2). Although Level 2 evidence supports the economic impact of RSV in young children in Singapore, the findings are based on a single retrospective analysis, which, despite covering a substantial patient population, may not fully capture the broader financial and healthcare implications. Future research incorporating real-world healthcare expenditure data, hospital-specific costs, and prospective cost-effectiveness analyses—potentially leveraging sources like the Singapore Household Expenditure Survey—would provide a more comprehensive assessment.

In summary, the burden of RSV disease in young children may be substantial, especially in those < 2 years old. The costs associated with RSV infections is likely underestimated in Singapore, mainly due to the absence of a national surveillance system, data on primary care consultations, long-term effects of RSV illness, and indirect costs. This substantial unmet medical need emphasizes an urgent need for RSV prevention in all infants in Singapore*.*

### Protection against RSV infections in infants

The high burden of disease and year-round RSV circulation pattern in Singapore emphasizes the need for appropriate preventive measures for all infants. Over the past two decades, the only product available to prevent RSV disease in high-risk infants was palivizumab, a monoclonal antibody approved in 1999 [43, 44]. Palivizumab targets the RSV-F glycoprotein and is administered in five monthly doses throughout the local RSV season because of its short half-life (20 days) [45]. However, it is recommended only for children at high risk of RSV disease, including premature infants and those with chronic lung disease (CLD), hemodynamically significant congenital heart disease (CHD), pulmonary abnormality, neuromuscular disease, or immunocompromised condition [28, 45]. This scenario, along with the high cost of palivizumab limits its wider accessibility [1, 46]. It is, therefore, imperative to include emerging preventive therapies for all infants against RSV through public programs. To that end, the faculty developed a consensus opinion on the efficacy and safety data of nirsevimab.

Nirsevimab is a potent, recombinant human immunoglobulin G1 monoclonal antibody that specifically targets antigenic site Ø on the prefusion RSV-F protein [4]. It neutralizes a diverse panel of RSV A and B strains with > 50-fold higher activity than palivizumab [47]. Furthermore, it contains a three amino acid YTE (M252Y/S254T/T256E) substitution in the Fc region, resulting in an extended half-life of mean 71 days in infants [48], which translates into durable protection of at least five months [49]. Nirsevimab was approved in the UK and Europe in 2022 for the prevention of RSV LRTI in neonates and infants during their first RSV season [50]. It was approved in the US, Australia, and Canada in 2023 for newborns and infants who were either born during or entering their first RSV season.

The approvals of nirsevimab were based on randomized, placebo-controlled, blinded clinical trials evaluating the efficacy and safety of nirsevimab for the prevention of RSV-associated medically attended (MA) acute LRTI in premature and term infants, as well as a pooled analysis of data from three clinical trials (Table 2) [51–53]. The MEDI8897 Phase IIb, multicenter trial randomized a total of 1453 preterm infants (≥ 29 to < 35 weeks gestation) entering their first RSV season (2:1) to receive a single intramuscular dose of 50 mg nirsevimab (*n* = 969) or placebo (*n* = 484) [51]. Infants with a history of CLD/CHD were excluded from this trial. At baseline, the mean age was 3.3 months, 53% were ≤ 3 months of age at randomization, and 62% were > 32 weeks gestational age at birth. The results showed that the incidence of MA RSV LRTIs through 150 days (primary endpoint) was 70.1% lower [95% confidence interval (CI): 52.3%–81.2%; *P* < 0.001] in the nirsevimab group compared with the placebo group. Furthermore, the incidence of hospitalizations due to RSV LRTI was reduced by 78.4% (95% CI: 51.9%–90.3%; *P* < 0.001) in those who received nirsevimab.

**Table 2:** Summary of key efficacy findings of nirsevimab from pivotal studies

Study and study phase	Patient population	Primary outcomes	Secondary outcomes
Phase 2b [51]	Preterm infants (≥ 29 to < 35 weeks), *n* = 1453	• MA RSV LRTI: 70.1% relative reduction (95% CI: 52.3%–81.2%; *P* < 0.001) vs. placebo	• Hospitalization due to RSV LRTI: 78.4% relative reduction (95% CI: 51.9%–90.3%; *P* < 0.001) vs. placebo
MELODY, phase III [52, 54]	Healthy late preterm and term infants (> 35 weeks), *n* = 3012	• MA RSV LRTI: 76.4% relative reduction (95% CI: 62.3%–85.2%; *P* < 0.001) vs. placebo	• Hospitalization due to RSV LRTI: 76.8% relative reduction (95% CI: 49.4%–89.4%) vs. placebo • Very severe RSV LRTI: 78.6% (95% CI: 48.8%–91.0%)
HARMONIE, phase IIIb [58]	Preterm infants (≥ 29 weeks), *n* = 8058	• RSV LRTI-associated hospitalization: 83.2% relative reduction (95% CI: 67.8%–92.0%; *P* < 0.001) vs. standard care	• Very severe RSV LRTI: 75.7% relative reduction (95% CI: 32.8%–92.9%; *P* = 0.004) vs. standard care
Pooled analysis (phase IIb and MELODY) [53]	Term and infants with ≥ 29 weeks gestation age, *n* = 2350	• MA RSV LRTI: 79.5% relative reduction (95% CI: 65.9%–87.7%; *P* < 0.001) vs. placebo	• Hospitalization due to RSV LRTI: 77.3% relative reduction (95% CI: 50.3%–89.7%; *P* < 0.001) vs. placebo
MEDLEY [57]	Infants with CLD or CHD and preterm infants with gestational age < 35 weeks, *n* = 925	• MA RSV LRTI: RRR 33.2% vs. palivizumab	

*CHD* congenital heart disease, *CI* confidence interval, *CLD* chronic lung disease, *LRTI* lower respiratory tract infection, *MA* medically-attended, *RRR* relative risk reduction, *RSV* respiratory syncytial virus

The pivotal phase III, multicenter, randomized controlled MELODY study included 3012 healthy late preterm and term infants (> 35 weeks’ gestation) entering their first RSV season, with 1998 infants in the nirsevimab group and 996 infants in the placebo group [52, 54]. The infants eligible for RSV immunoprophylaxis with palivizumab based on local or national guidelines, were excluded. At baseline, 59% of infants were ≤ 3 months of age at randomization and 88% were born at term [54]. The efficacy of nirsevimab administration against MA RSV LRTIs through 150 days after injection (primary endpoint) was 76.4% (95% CI: 62.3%–85.2%; *P* < 0.001), with no evidence of waning efficacy. Efficacy against RSV LRTI hospitalization was 76.8% (95% CI: 49.4%–89.4%), and efficacy against very severe RSV LRTI was 78.6% (95% CI: 48.8%–91.0%) [54]. Children (*N* = 2911) from the MELODY trial were followed through their second season without redosing [55]. The results showed no increase in the incidence of MA LRTI of any cause or hospitalization for respiratory illness of any cause, suggesting that prophylaxis with nirsevimab during the first season does not lead to an increased burden of disease in the second year of life. Notably, in South Africa where there was no RSV circulation in the five months following the start of MELODY, an analysis of MA LRTI after 360 days revealed a relative risk reduction of 50% with nirsevimab administration, thus indicating protection beyond the five-month study period.

To determine the efficacy of nirsevimab under a weight-banded dosing regimen, a pooled analysis of 2350 infants born between 29 weeks gestational age and full term was conducted [53]. This comprised 860 preterm infants (≥ 29 to < 35 weeks gestation) weighing < 5 kg from the phase 2b trial, and 1490 term or late preterm infants (≥ 35 weeks gestation) from the MELODY trial. Compared to placebo, a single weight-banded dose of nirsevimab led to 79.5% (95% CI: 65.9%–87.7%) relative risk reduction (RRR) of MA RSV LRTI (primary efficacy endpoint). Similar efficacy was reported for the secondary efficacy endpoints, with RRRs of 77.3% (95% CI: 50.3%–89.7%) against hospital admission for MA RSV LRTI and 86.0% (95% CI: 62.5%–94.8%) against very severe RSV disease. Compared to baseline, defined as pre-nirsevimab dose and hence assumed to be maternal RSV antibodies in infants [87 IU/mL (95% CI: 79–95) in the phase 2b study and 134 IU/mL (95% CI: 125–143) in the MELODY study], nirsevimab recipients had RSV-neutralizing antibody levels at day 151 > 50-fold than baseline and this remained > sevenfold higher at day 361, suggesting a significant extended protection up to one-year post-dose comparable to target levels [56].

In assessing nirsevimab for infants at high risk of RSV LRTIs, the panel examined results from the MEDLEY and MUSIC studies. In MEDLEY, a multicenter trial, 925 infants at higher risk for severe RSV disease, including infants with CLD or CHD and preterm infants with gestational age < 35 weeks entering their first RSV season were randomized 2:1 to receive either a single dose of nirsevimab (50 mg if < 5 kg body weight or 100 mg if ≥ 5 kg body weight at the time of dosing) or five monthly intramuscular doses of 15 mg/kg palivizumab [57]. There was a RRR of 33.2% in MA RSV LRTI in infants receiving nirsevimab compared to palivizumab (4/616 in the nirsevimab group and 3/309 in the palivizumab group).

Finally, in the open-label pragmatic HARMONIE phase IIIb, multicenter trial where 8058 infants (aged ≤ 12 months, born at gestational age ≥ 29 weeks) received nirsevimab or standard care when they were entering their first RSV season or at birth (if born during the RSV season), an effectiveness of 83.2% (95% CI: 67.8%–92.0%; *P* < 0.001) for nirsevimab against RSV LRTI-associated hospitalizations was observed [58]. Additionally, very severe RSV LRTIs occurred in 0.1% (*n* = 5) infants in the nirsevimab group, compared to 0.5% (*n* = 19) infants in the standard-care group, resulting in a nirsevimab efficacy of 75.7% (95% CI: 32.8%–92.9%; *P* = 0.004). This study showed that nirsevimab protected infants against RSV under conditions close to real-world settings.

Collectively, a meta-analysis by Ricco et al., including 45,238 infants across five RCTs, seven real-world studies and one health report revealed that nirsevimab had a pooled immunization efficacy of 88.4% (95% CI: 84.7%–91.2%) in preventing RSV-related hospital admissions. Heterogeneity assessment revealed moderate heterogeneity (*I*^2^ = 24.3%), thus reflecting consistency between RCT efficacy and real-world effectiveness. In summary, the analysis concluded that nirsevimab was effective in preventing hospital admissions due to LRTDs [59].

Pooled safety data from the nirsevimab clinical trials revealed a favorable safety profile, with rash (0.7%) occurring within 14 days post-dose, pyrexia (0.5%), and injection site reactions (0.3%) within seven days post-dose being the most frequent adverse reactions [50]. A recent summary of safety data including three pivotal studies (MELODY, MEDLEY, and phase IIb) indicated that the incidence, severity, and nature of adverse events (AEs) were comparable across nirsevimab, placebo, and palivizumab treatments [60]. In the phase III MELODY and phase IIb trial in healthy term and preterm infants, only a few cases in the nirsevimab and placebo arms experienced an adverse event considered to be related to the trial regimen (MELODY: 1.0% vs. 1.4%; phase IIb: 2.3% vs. 2.1%); none were considered serious [51, 52]. The postbaseline anti-drug antibodies were detected in 5.6% (52/929) of recipients of nirsevimab and 3.8% (18/469) of placebo recipients at day 361 in the phase IIb trial [51]. Similar rates of adverse events between nirsevimab and the control group were also observed in studies in high-risk (MEDLEY) and immunocompromised (MUSIC) populations [61].

Overall, the studies showed that the administration of a single dose of nirsevimab was highly effective [59] against RSV-associated hospitalization and MA LRTIs due to RSV in infants entering their first RSV season. These findings support the recommendation by the expert panel that nirsevimab has a good safety profile and is effective in preventing RSV LRTIs and hence may be administered to all infants in Singapore before or during their first RSV season (Table 1, Statement 5; Level of evidence 1).

### Implementation of nirsevimab as a public health measure: programs implemented globally and how they can be applied in Singapore

#### Implementation of nirsevimab to reach all infants: US

Following the US FDA approval of nirsevimab in 2023, the Centers for Disease Control (CDC) and Prevention and the Advisory Committee on Immunization Practices (ACIP) recommended nirsevimab for infants < 8 months of age who are born during or entering their first RSV season, and infants and children 8–19 months of age who are at an increased risk for severe RSV disease and entering their second RSV season [46, 62]; the American Academy of Pediatrics also endorsed this recommendation [63]. The ACIP also recommended that infants born during the RSV season should receive their first dose within their first week of life, ideally before hospital discharge or at the first outpatient visit [64]. A static decision-analytic model of the US birth cohort to determine nirsevimab’s impact on RSV-related health events and costs estimated 529,915 cases of MA RSV LRTIs in the first RSV season from US birth cohort’s, affecting 14% of the infants [(1) term and preterm infants, (2) preterm infants not eligible for palivizumab, (3) palivizumab-eligible infants] [65]. With 71% and 80% uptake rate of a universal immunization program in term/preterm infants and palivizumab-eligible infants, respectively, nirsevimab use was estimated to result in 290,174 fewer MA RSV LRTIs, 53% reduction in hospital admissions, and 55% reduction in emergency room and primary cost events, resulting in a US dollar 612 million decrease in expenditure arising from a 55% reduction in health events. Following the approval of nirsevimab and its implementation in an immunization program, the New Vaccine Surveillance Network assessed 699 infants hospitalized with acute respiratory infections from October 2023 to February 2024 across four US sites [62]. Early estimates showed nirsevimab was effective in reducing 90% of RSV-associated hospitalizations in infants in their first RSV season. The results of the additional studies are summarized in Table 3 [66].

**Table 3:** Summary of the effectiveness of nirsevimab from pivotal studies

Country/study	Effectiveness in reducing RSV hospitalization	Effectiveness in preventing ICU admissions
USA (VISION) [66]	98% (95% CI: 95%–99%)	Not reported
USA (NVSN) [66]	91% (95% CI: 79%–96%)	Not reported
Spain—Galicia [70]	82% (95% CI: 65.6%–90.2%)	Not reported
Spain—Cataluña [76]	87.6% (95% CI: 82.1%–91.4%)	90.1% (95% CI: 76.3%–95.9%)
Spain—Murcia, Valencia, Valladolid [71]	84.4% (95% CI: 76.8%–90.0%)	Not reported
Spain—Castellon [73]	RR 0.16 (for children 0–5 months)	Not reported
Spain—nationwide [75]	71% (for catch-up immunization)	Not reported
	78% (for at-birth immunization)	
Spain—Navarre [77]	88.7% (95% CI: 69.6%–95.8%)	85.9% (95% CI: 13.2%–97.7%)
Luxembourg [81]	69%	Not reported
France (ENVIE) [78]	83.0% (95% CI: 73.4%–89.2%)	Not reported
France [79]	75.9%	

*CI* confidence interval, *ICU* intensive care unit, *RR* relative risk, *RSV* respiratory syncytial virus

#### Implementation of nirsevimab to reach all infants: Spain

Following the European Medicines Agency approval in 2022, Spain included nirsevimab in the national immunization program for the 2023–2024 season [67]. The Spanish National Immunization Technical Advisory Group recommended nirsevimab in the routine immunization schedule for newborns and infants aged < 6 months, with annual administration in children aged < 2 years with underlying disease [68].

The immunization campaign in Galicia for the 2023/24 season using nirsevimab (NIRSE-GAL study) ran from September 25, 2023, to March 31, 2024, aligning with the local RSV season [69]. The Galicia region was considered due to its digitalized healthcare records. The immunization coverage was 91.7% for infants born during the season, with 9408 infants immunized. This study showed a reduction of RSV hospitalization cases of 89.8% (interquartile range 87.5%–90.3%) and a number needed to immunize (NNI) of 25 (IQR 24–32) (Table 3) [70]. Another multicenter hospital-based active surveillance in nine hospitals across three autonomous regions in Spain (five in Valencia, three in Murcia, and one in Valladolid, Castilla y León) included 15,676 infants eligible for nirsevimab, representing 6.4% of the eligible Spanish infant population (Table 3) [71]. Nirsevimab was effective in preventing 84.4% of RSV-LRTI hospitalizations, with population-based coverage ranging between 78.7% and 98.6% depending on the hospital. Following the results of nirsevimab effectiveness in Galicia and Valencia, the introduction of nirsevimab during the 2023–2024 season in Catalonia was associated with a 40% reduction in all-cause bronchiolitis risk in infants under 12 months compared to the previous season (62% vs. pre-pandemic) and 76% reduction in RSV infections [72]. Additionally, systematic nirsevimab immunization during the 2023–2024 season in the Castellon province in Spain significantly reduced RSV hospitalization risk in infants under six months [relative risk (RR) 0.16], while its impact was less pronounced in older age groups (Table 3) [73]. A recent study using data from a large primary care network (MEDIPRIM) in Spain found that nirsevimab was effective in preventing MA RSV-LRTIs in infants (*n* = 160), especially the catch-up cohort (*n* = 128) (75.8% overall; 80.2% in catch-up infants), thus highlighting the importance of catch-up immunization program [74]. Another nationwide case–control study in Spain during the 2023/24 season found that nirsevimab was effective (80% per protocol) in preventing 71% of RSV-related hospitalizations with catch-up immunization and 78% of hospitalizations (83% per protocol) with at-birth immunization, with similar effectiveness for ICU admissions and mechanical ventilation (Table 3). However, a slightly lower effectiveness (60%–70%) was reported in preterm and low-birthweight infants [75]. The studies on effectiveness of nirsevimab conducted in Spain are summarized in Table 3 [76, 77].

#### Implementation of nirsevimab to reach all infants: France

Nirsevimab was approved in France in 2023 and a single dose is recommended free of charge by French medical societies to all children born after February 2023. Because of shortages occurring during the national campaign, nirsevimab was then preferentially given to newborns and young infants, as well as young infants under the age of one year who were palivizumab-eligible. A prospective, multicenter, matched case–control study (ENVIE) following the implementation of the national campaign revealed 83.0% effectiveness (95% CI: 73.4%–89.2%) of nirsevimab against bronchiolitis-associated hospitalization (Table 3). In addition, nirsevimab reduced RSV requiring intensive care by 69.6% (95% CI: 42.9%–83.8%) and RSV requiring respiratory assistance by 67.2% (95% CI: 38.6%–82.5%) [78]. Data from a network of pediatric ICUs reported nirsevimab effectiveness of 75.9% in infants who received nirsevimab ≥ 8 days before hospitalization (Table 3), and 80.4% (95% CI: 61.7%–89.9%) when adjusted for all infants who received nirsevimab regardless of time between administration of treatment and pediatric ICU admission [79].

#### Implementation of nirsevimab to reach all infants: Italy and Luxembourg

The implementation of universal RSV prophylaxis with nirsevimab in Valle d’Aosta, Italy during the 2023–2024 RSV season was associated with a significant reduction in RSV bronchiolitis hospitalizations (3.2% vs. 7% in the previous season, *P* < 0.001), with no hospitalizations among treated infants [80]. Similarly, following the introduction of nirsevimab immunization in Luxembourg (84% neonatal coverage in 2023), RSV-related hospitalizations, ICU admissions, and hospital stay durations significantly decreased, particularly in infants under six months, suggesting reduced RSV severity and healthcare burden (Table 3) [81].

#### Optimization of nirsevimab implementation in Singapore

On the basis of its clinical trial results and the success of nirsevimab implementation in national RSV immunization programs described earlier, the panel members agreed that the introduction of nirsevimab into a national immunization program for all infants in Singapore would alleviate the burden of RSV disease (Table 1, Statement 6; Expert opinion). However, the panel members suggested that while this is a clinical strategy for RSV prevention, the inclusion of nirsevimab in the national childhood immunization program would require economic evaluation and public health prioritization. As the RSV season in Singapore extends over about seven months but can vary each year, immunizing all infants at or soon after birth can be a feasible option for protection during the most vulnerable first 5–6 months of life. The evidence for nirsevimab protection extending up to a year would also be desirable where seasonality is prolonged, such as in Singapore. The acceptance of nirsevimab newborn immunization in Singapore is likely to be high, as reflected by the vaccine coverage rates of other pediatric vaccines. The flexible dosing of nirsevimab may also allow for a tailored approach where parents may choose an outpatient setting for vaccinating their infant. Extending the RSV surveillance system and conducting postimplementation real-world effectiveness studies in Singapore can guide refinement of the RSV seasonality and annual immunization campaigns in the future. To ensure high coverage of infants and effective implementation of nirsevimab immunization in Singapore, strategies such as stakeholder engagement, public health funding, targeted communication campaigns, and clear service protocols—similar to those successfully used in Spain, France, and the US—should be considered. The success of RSV immunization in infants in Singapore is also likely to guide the public healthcare policy of other countries in the region that share the same tropical climate. Furthermore, while all infants should be immunized within their first year, the necessity of a second dose of nirsevimab beyond their first year remains uncertain but may be prioritized in high-risk infants (Table 1, Statement 7; Expert opinion). In the high-risk group who remains susceptible to MA LRTI or hospitalization, the MEDLEY study demonstrated that a second dose results in high neutralizing antibody levels sufficient to confer protection, hence our opinion is consistent with guidance by other recommendation bodies as well [55, 64, 82].

There are two other options registered in Singapore for protecting against RSV infections: palivizumab and maternal immunization RSV vaccine. Palivizumab has been available in Singapore since 2016, but it is not widely accessible due to its high costs and licensed indication. Prior to the potential future inclusion of nirsevimab in the national childhood immunization program, the advisors suggested that nirsevimab should be positioned as a replacement for palivizumab based on the results of the MEDLEY, MUSIC, and MELODY studies, encompassing preterm infants, infants with cardiovascular, respiratory, or oncological comorbidities, those on long-term immunosuppressive therapy, and other high-risk groups. A single dose of nirsevimab would also provide greater convenience than the five monthly palivizumab doses required for protection across the RSV season.

The maternal immunization RSV vaccine (RSVpreF) has been registered in Singapore since July 2024. It is to be administered between 32 and 36 weeks of pregnancy [83]. The mechanism of action consists of transplacental transfer of maternal immunoglobulin G, with transfer peaking in the last four weeks of pregnancy, and greater efficiency when the interval between immunization and delivery is ≥ 30 days [84]. Fetus antibody titers appear about two weeks following maternal vaccination [1]. A phase III study shows vaccine efficacy against MA RSV LRTIs at 90 days of 57.1% and 67.7% (99.17% CI, 15.9% to 89.5%) for RSV-associated hospitalization [85]. The monthly results showed a decline in efficacy over time, with a vaccine efficacy at six months of 51.3% for MA RSV LRTI and a 56.8% efficacy against RSV hospitalizations.

Safety data indicated more early-term pregnancies in the vaccine group than the placebo, with concerns about wide CIs and insufficient data for policy decisions [86]. This led to FDA’s decision of indication over the 32–36 weeks of pregnancy [87]. The FDA has requested for additional studies in pregnant people to assess preterm birth and hypertensive disorders of pregnancy, including preeclampsia [88]. RSVPreF3-Mat, another maternal RSV vaccine reached the late stages of clinical development. However, its development was terminated in 2022 because preterm birth and neonatal death rates were higher in the vaccine versus the placebo group in the phase III safety and efficacy trial [89].

Another emerging option for RSV protection includes clesrovimab (MK-1654), a long-acting human IgG1 monoclonal antibody that targets the highly conserved antigenic site IV of the RSV-F glycoprotein, with activity against both pre-F and post-F conformations [90]. A phase Ib/IIa double-blind study in 183 healthy preterm and full-term infants two weeks to eight months of age showed that clesrovimab was generally well tolerated with a geometric mean apparent half-life of 44.9 days [91]. Currently, two phase III trials are underway.

In summary, the efficacy and safety data from the pivotal trials of nirsevimab is reflected in the real-world evidence from national immunization programs implemented elsewhere. These positive health outcomes are likely to be reflected in future newborn vaccination programs implemented in Singapore, where there is a variable and prolonged seasonality. The infants in their first year of life need to be prioritized, and a second dose of nirsevimab for infants entering their second RSV season needs further evaluation but can be given to high-risk groups if assessed to be more cost-effective than palivizumab.

## Conclusions

RSV substantially contributes to the morbidity and mortality in children aged < 2 years in Singapore, particularly during their first year of life. This evidence-based consensus recommends that the administration of a single dose of nirsevimab may be offered to infants at birth for the prevention of RSV in Singapore.

## Supplementary Information

Below is the link to the electronic supplementary material.

Supplementary file1 (DOCX 22 KB)

## Data Availability

Data sharing not applicable to this article as no datasets were generated or analyzed during the current study.
