# Supplementary material for: Expert consensus on the burden of respiratory syncytial virus disease and the utility of nirsevimab for disease prevention and protection of infants
Source: World J Pediatr. 2025 Jun 28;21(6):552–65. doi: 10.1007/s12519-025-00926-2 (PMC12627213; doi:10.1007/s12519-025-00926-2)
Supplement: Supplementary file 1 — Supplementary file1 (DOCX 22 KB) [file 12519_2025_926_MOESM1_ESM.docx]

**Supplementary tables**

**Supplementary Table 1 Search string and MeSH terms used for literature search**

| Search string | (((Respiratory Syncytial Virus Infection[MeSH Terms]) OR (((((((infant[MeSH Terms]) OR (infant, newborn[MeSH Terms])) OR (neonate[MeSH Terms])) OR (child[MeSH Terms])) OR (infant, premature[MeSH Terms])) OR (preterm infants[MeSH Terms])) OR (pediatric))) OR ((((((((((((((((((((((((((Respiratory tract infection[MeSH Terms]) OR (Respiratory infection[MeSH Terms])) OR (Hospitalization[MeSH Terms])) OR (Length of stay[MeSH Terms])) OR (Length of stay[MeSH Terms])) OR (Hospital stay[MeSH Terms])) OR (Mechanical ventilation[MeSH Terms])) OR (Continuous positive airway pressure[MeSH Terms])) OR (CPAP ventilation[MeSH Terms])) OR (Bronchiolitis[MeSH Terms])) OR (Bronchiolitis, viral[MeSH Terms])) OR (Pneumonia, viral[MeSH Terms])) OR (Emergency room visit[MeSH Terms])) OR (Intensive care unit, pediatric[MeSH Terms])) OR (Intensive care unit, neonatal[MeSH Terms])) OR (QALYs[MeSH Terms])) OR (Quality-Adjusted Life years[MeSH Terms])) OR (LRTI)) OR (Medically attended lower respiratory tract infection)) OR (supplemental oxygen)) OR (safety[MeSH Terms])) OR (wheez*)) OR (otitis[MeSH Terms])) OR (asthma[MeSH Terms])) OR (adverse event)) OR (Adverse Drug Reaction[MeSH Terms]))) AND ((((((((nirsevimab) OR (beyfortus)) OR (MED-18897)) OR (Monoclonal antibody MED-18897)) OR (MED 18897)) OR (Monoclonal antibody MED 18897)) OR (Antibodies, monoclonal, humanized MED 18897)) OR (Antibodies, monoclonal, humanized MED-18897)) |
| --- | --- |
| MeSH terms | Nirsevimab; Beyfortus; antibodies; monoclonal MED 18897; antibodies; monoclonal, humanized MED 18897; MED 18897; MED-18897; respiratory syncytial virus; respiratory syncytial virus, human; respiratory syncytial viruses; infant; infant, newborn; neonate; child; infant, premature; preterm infants; respiratory tract infection; respiratory infection; hospitalization; length of stay; hospital stay; mechanical ventilation; ventilation, mechanical; continuous positive airway pressure; CPAP ventilation; bronchiolitis; bronchiolitis, viral; pneumonia, viral; emergency room visit; intensive care unit, pediatric; intensive care unit, neonatal; QALYs; Quality-Adjusted Life years; safety; adverse drug reaction; otitis; asthma |

*Asterisks in the search string indicate wildcard symbols that were used to broaden the search.

**Supplementary Table 2 Oxford Centre for Evidence-Based Medicine 2011 levels of evidence**

| **Question** | Step 1 (Level 1^a^) | Step 2 (Level 2^a^) | Step 3 (Level 3^a^) | Step 4 (Level 4^a^) | Step 5 (Level 5) |
| --- | --- | --- | --- | --- | --- |
| **How common is the problem?** | Local and current random sample surveys (Or censuses) | Systematic review or surveys that allow matching to local circumstances^b^ | Local non-random sample^b^ | Case-series^b^ | n/a |
| **Is this diagnostic or monitoring test accurate** (Diagnosis) | Systematic review of cross-sectional studies with consistently applied reference standard and blinding | Individual cross-sectional studies with consistently applied reference standard and blinding | Non-consecutive studies, or studies without consistently applied reference standards^b^ | Case-control studies, or poor or non-independent reference standard^b^ | Mechanism-based reasoning |
| **What will happen if we do not add a therapy?** (Prognosis) | Systematic review of cohort studies | Inception of cohort studies | Cohort study or control arm of randomized trial^a^ | Case-series or case-control studies, or poor-quality prognostic cohort study^b^ | n/a |
| **Does this intervention help?** (Treatment benefits) | Systematic review of randomized trials or *n*-of-1 trials | Randomized trial or observational study with dramatic effect | Non-randomized controlled cohort/follow-up study^b^ | Case-series, case-control studies, or historically controlled studies^b^ | Mechanism-based reasoning |
| **What are the COMMON harms?** (Treatment harms) | Systematic review of randomized trials, systematic review of nested case-control studies, *n*-of-1 trial with the patient you are raising the question about, or observational study with dramatic effect | Individual randomized trial or (exceptionally), observational study with dramatic effect | Non-randomized controlled cohort/follow-up study (post-marketing surveillance) provided there are sufficient numbers to rule out a common harm. (For long-term harms the duration of follow-up must be sufficient)^b^ | Case-series, case-control, or historically controlled studies^b^ | Mechanism-based reasoning |
| **What are the RARE harms?** (Treatment harms) | Systematic review of randomized trials or *n*-of-1 trials | Randomized trial or (exceptionally) observational study with dramatic effect |  | Case-series, case-control, or historically controlled studies^b^ | Mechanism-based reasoning |
| **Is this (early detection) test worthwhile?** (Screening) | Systematic review of randomized trials | Randomized trial | Non-randomized controlled cohort/follow-up study^b^ | Case-series, case-control, or historically controlled studies^b^ | Mechanism-based reasoning |

^a^ Level may be graded down on the basis of study quality, imprecision, indirectness (study PICO does not match questions PICO), because of inconsistency between studies, or because the absolute effect size is very small; Level may be graded up if there is a large or very large effect size.
^b^ As always, a systematic review is generally better than an individual study.

**Supplementary Table 3 Pre-meeting survey (round 1 Delphi) results**

| **Statements rated based on an agreement scale from 1 to 5** | **Agreement rating (%)** |
| --- | --- |
| RSV is a major burden of disease among children under 1 year old in Singapore | 100% |
| All infants need to be protected from RSV | 89% |
| RSV is a major cause of hospitalization due to bronchiolitis and pneumonia in children under 1 year old in Singapore | 100% |
| RSV infections follow a peak season from May to September, with constant circulation throughout the year in September | 67% |
| RSV contributes to significant healthcare costs (direct and indirect) as well as psychological stress to parents | 55.6% |
| There is an urgency to prevent RSV in all infants in Singapore. | 89% |
| **Statements with open-ended discussion** | **Expert opinion** |
| Key attributes in the selection of a preventative strategy against RSV for all infants in Singapore | 1. Well-established safety profile 2. Established efficacy for all infants 3. Able to provide protection beyond 5 months 4. Able to be administered all year long 5. Able to be administered at birth |
| Preferred preventative strategy against RSV based on the published evidence | 1. Nirsevimab long-acting mAb (*n* = 4) 2. Palivizumab short-acting mAb (*n* = 1) 3. Maternal immunization (*n* = 1) 4. Supportive care (*n* = 0) 5. Other (*n* = 3) |

*mAb* monoclonal antibody, *RSV* respiratory syncytial virus
